# Supplementary figures and images for: Deconstruction of the Ras switching cycle through saturation mutagenesis
Source: eLife. 2017 Jul 7;6:e27810. doi: 10.7554/eLife.27810 (PMC5538825; doi:10.7554/eLife.27810)

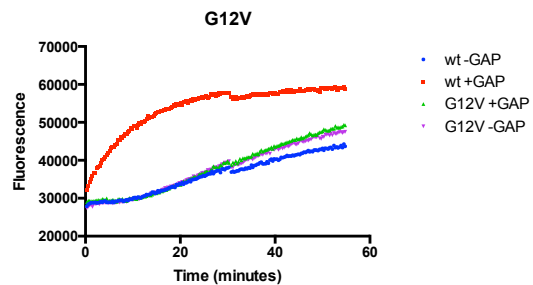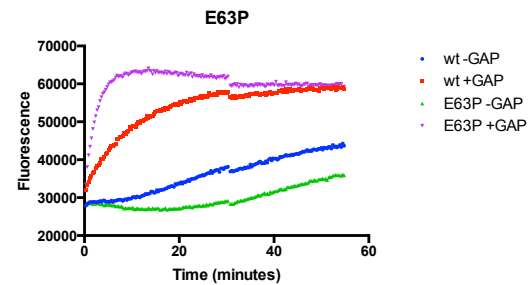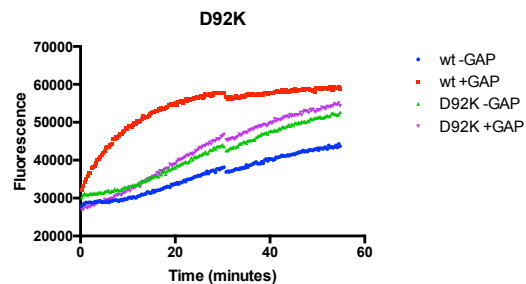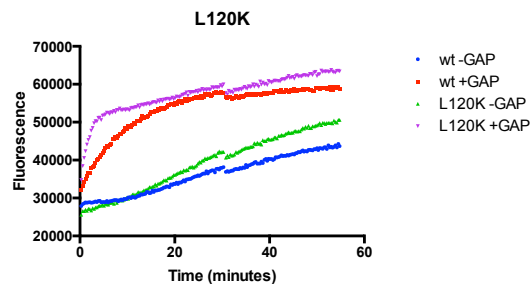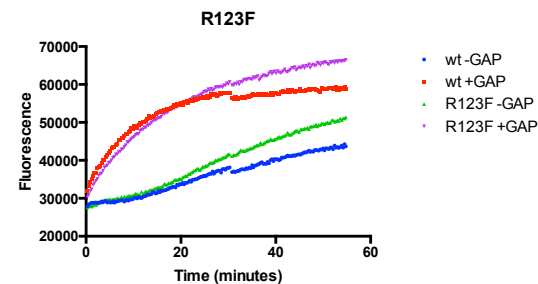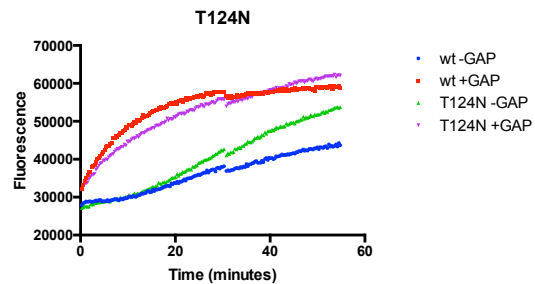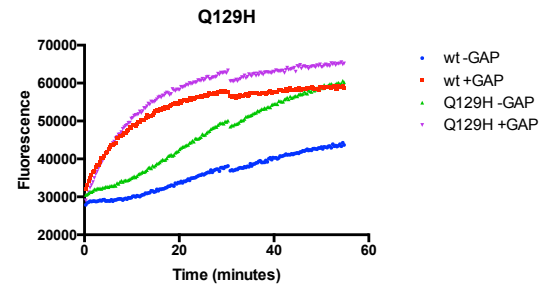

Supplement: Figure 2—source data 1. — DOI: http://dx.doi.org/10.7554/eLife.27810.009 [file elife-27810-fig2-data1.pdf]
