# Supplementary material for: Deconstruction of the Ras switching cycle through saturation mutagenesis
Source: eLife. 2017 Jul 7;6:e27810. doi: 10.7554/eLife.27810 (PMC5538825; doi:10.7554/eLife.27810)
Supplement: Supplementary file 2. — DOI: http://dx.doi.org/10.7554/eLife.27810.028 [file elife-27810-supp2.docx]

| **Data Collection** | | | | | |
| --- | --- | --- | --- | --- | --- |
|  | Human WT  Ras.GMPPNP (RT) | Human L120A  Ras.GMPPNP (LT) | Human L120A  Ras.GMPPNP (RT) | Choano  Ras.GTP  (LT) | Choano  Ras.GDP  (LT) |
| Temperature | 277K | 100K | 277K | 100K | 100K |
| Wavelength (Å) | 0.88557 | 1.000 | 0.88557 | 1.11587 | 1.000 |
| Space group | H32 | H32 | H32 | P2_1_ | P 4_3_ 2_1_ 2 |
| Cell dimensions  a, b, c (Å)  α, β, γ ˚ | 90.0 90.0 136.7  90 90 120 | 87.96, 87.96, 133.1  90.0, 90.0 120.0 | 90.0 90.0 136.0  90 90 120 | 38.6, 119.2, 38.6  90.0, 117.3, 90.0, | 67.2, 67.2, 184.7  90, 90, 90 |
| Resolution (Å)  (high res shell) | 45.58 - 1.65 (1.68 - 1.65) | 50-1.25  (1.27-1.25) | 45.32 - 1.35 (1.37 - 1.35) | 39.70 - 1.60 (1.63 - 1.60) | 47.53 - 1.85 (1.89 - 1.85) |
| Rmeas (%)  Rpim (%) | 17.9 (327.2)  4.0 (75.6) | 4.5 (59.4)  1.0 (30.8) | 9.9 (297.2)  3.2 (94.2) | 9.8 (94.4)  4.7 (55.7) | 9.3 (143.7)  2.7 (51.1) |
| *I/σ(I)* | 12.6 (1.2) | 36.9 (2.4) | 16.2 (1.2) | 9.5 (1.4) | 12.5 (2.0) |
| *CC_1/2_* | 0.999 (0.514) | 1.00 (0.790) | 0.999 (0.565) | 0.997 (0.428) | 0.995 (0.923) |
| Completeness (%) | 100 (100) | 99.0 (92.0) | 100 (100) | 99.9 (99.8) | 100.0 (100.0) |
| Multiplicity | 19.8 (18.6) | 18.7 (4.6) | 19.7 (20.0) | 4.5 (3.8) | 9.9 (8.8) |
| Wilson B factor | 17.4 | 11.5 | 14.6 | 12.1 | 25.7 |
| **Refinement** | | | | | |
| Reflections used | 25866 (2838) | 54265 (2483) | 46639 (4612) | 40548 (2634) | 37071 (2786) |
| Rfree reflections | 1254 (121) | 2731 (123) | 2725 (132) | 2065 (137) | 1837 (135) |
| ­R_work_/R_free_ | 14.16/16.84 (25.26/28.43) | 14.11/ 15.24  (23.49/25.27) | 14.50/15.36 (27.20/26.87) | 22.85/27.98  (30.00/36.71)  16.64/20.58*  (26/29)* | 21.62/24.90  (35.8/35.6) |
| Twinning fraction |  |  |  | 0.47 for l, –k, h |  |
| Number of non hydrogen atoms | 1695 | 1782 | 1728 | 3010 | 3026 |
| Protein | 1530 | 1570 | 1578 | 2754 | 2778 |
| Ligands | 36 | 46 | 36 | 68 | 70 |
| **Average B factors** | | | | | |
| Overall | 28.39 | 21.65 | 26.7 | 20.0 | 41.9 |
| Protein | 27.80 | 20.15 | 26.4 | 18.8 | 41.7 |
| Ligand | 18.99 | 22.40 | 15.6 | 12.4 | 42.3 |
| Solvent | 38.0 | 35.62 | 34.7 | 22.8 | 45.5 |
| Root mean square deviation from ideality | | | | | |
| Bonds (Å) | 0.012 | 0.011 | 0.009 | 0.011 | 0.006 |
| Angles (Å) | 1.17 | 1.12 | 1.052 | 1.23 | 0.885 |
| Ramachandran Statictics | | | | | |
| Favored (%) | 98.8 | 96.95 | 97.6 | 98.8 | 98.8 |
| Disallowed (%) | 0.0 | 0.61 | 0.0 | 0.0 | 0.3 |
| MolProbity clash score | 3.57 | 4.7 2 | 2.21 | 3.25 | 3.02 |
